# Transcriptome and MicroRNA Analysis of *Juglans regia* in Response to Low-Temperature Stress

**DOI:** 10.3390/ijms26041401

**Published:** 2025-02-07

**Authors:** Haochang Zhao, Xia Luo, Caihua Guo, Zhongrong Zhang, Kai Ma, Jianxin Niu, Shaowen Quan

**Affiliations:** 1 College of Agriculture, Shihezi University, Shihezi 832003, China; zhaohaochang@stu.shzu.edu.cn (H.Z.); luoxia@stu.shzu.edu.cn (X.L.); gch9512@163.com (C.G.); zhangzhongrong@xjshzu.com (Z.Z.); 2 Xinjiang Production and Construction Corps Key Laboratory of Special Fruits and Vegetables Cultivation Physiology and Germplasm Resources Utilization, Shihezi 832003, China; 3 Institute of Horticultural Crops, Xinjiang Academy of Agricultural Sciences, Urumqi 830091, China; sunshine002mk@163.com

**Keywords:** *Juglans regia*, low-temperature stress, RNA-seq, microRNA

## Abstract

Walnuts are among the globally significant woody food and oil tree species. At high latitudes, they frequently experience late-frost damage, inducing low-temperature stress, which significantly affects walnut seedlings. The aim of this study was to investigate the physiological and biochemical alterations in walnut seedlings under low-temperature (LT) stress along with its underlying molecular mechanisms. Physiological indices were determined, and the transcriptome and miRNA were sequenced by sampling leaves (0 h, 6 h, 12 h, 24 h, and 48 h) of two-month-old live seedlings of walnuts treated with a low temperature of 4 °C. The results indicated that LT stress induced an increase in electrical conductivity and malondialdehyde content while simultaneously causing a reduction in Fv/Fm. From the transcriptome comparison between the control and treated groups, a total of 12,566 differentially expressed genes (DEGs) were identified, consisting of 6829 up-regulated and 5737 down-regulated genes. Gene Ontology (GO) and Kyoto Encyclopedia of Genes and Genomes (KEGG) enrichment analyses revealed that the DEGs were primarily enriched in polysaccharide metabolic processes, responses to abscisic acid and phenylpropanoid biosynthesis pathways. Furthermore, the miRNA database identified 1052 miRNAs in response to low-temperature stress in walnuts; these miRNAs were found to target 7043 predicted genes. Through the integration and analysis of transcriptome and miRNA data, 244 differential DEGs were identified. Following GO and KEGG enrichment analyses of the differential target genes, we identified that these genes primarily regulate pathways involved in starch and sucrose metabolism, glyoxylate and dicarboxylate metabolism, and glycerophospholipid biosynthesis, as well as phenylalanine, tyrosine, and tryptophan biosynthesis, in walnut leaves under LT stress. Additionally, we conducted an in-depth analysis of the associations between differentially expressed genes (DEGs) and differentially expressed microRNAs (DEMs) within the starch and sucrose metabolism pathway. Real-time fluorescent quantitative PCR (qRT-PCR) validation of the expression patterns of a subset of differential genes confirmed the accuracy of the transcriptome data. This study unveils the potential molecular mechanisms underlying walnut’s response to low-temperature stress, providing valuable genetic resources for future research on the cold tolerance mechanisms of walnut in response to late-frost damage.

## 1. Introduction

Walnuts (*Juglans regia* L.), growing on a perennial deciduous tree from the Juglandaceae family, are one of the world’s four major dry fruits (alongside almonds, chestnuts, and cashews) and a key species for woody oilseed production in China, with significant economic value. Walnut kernels are abundant in a variety of nutrients, including unsaturated fatty acids and mineral elements, and possess health benefits such as anti-aging and immune enhancement properties [1]. China is the largest global producer of walnuts, followed by the United States, Iran, Turkey, Romania, Ukraine, France, and India [2]. The Xinjiang region is a major walnut production area in China. However, spring frosts and sudden temperature fluctuations have posed serious risks to its cultivation.

Low-temperature stress is a crucial environmental factor that limits plant growth and production, especially in temperate regions with frequent temperature fluctuations [3]. Plants respond to cold stress through a cascade of physiological and biochemical reactions [4], including increased osmotic and oxidative stress, which leads to membrane damage and protein dysfunction [5]. At the molecular level, this involves transcriptional regulation by transcription factors and the activation of functional proteins [6]. Many of these physiological and biochemical changes are mediated by the inter-regulation of various relevant functional genes induced by low temperatures [7]. While some key regulatory pathways and related genes have been studied in the model plant *Arabidopsis thaliana* [8], the molecular mechanisms underlying walnut’s response to low-temperature stress remain unclear.

With the advancement of genomics technology, transcriptomic approaches have been widely adopted to screen DEGs and analyze related functional mechanisms [9]. RNA-seq is particularly useful in plant transcriptome analysis due to its high efficiency, cost-effectiveness, and reliability, providing a convenient and rapid platform for large-scale transcriptome analysis [10]. In the last decade, RNA-seq has been extensively used in studies of the response of many plants to cold stress, including *Amygdalus persica* [11], *Camellia* [12], and *Prunus* [13], demonstrating its potential as a powerful tool in plant genetics research [14].

MicroRNAs (miRNAs) are small RNA molecules (19–25 nucleotides) that regulate gene expression by silencing target mRNAs post-transcriptionally [15]. A single miRNA can target hundreds of mRNAs, affecting the expression of many genes commonly involved in functional interaction pathways. First reported in nematodes (*Caenorhabditis elegans*) in 1993 [16], miRNAs in plants were also discovered in 2002 [17]; miRNAs have emerged as an important class of post-transcriptional gene regulators in plants and animals [18]. Studies have shown that miRNAs play important regulatory roles in plant response to abiotic stresses, including the regulation of growth and development, hormone secretion, signal transduction, and reducing damage due to environmental stresses [19]. For example, the *miR397-LACs* module modifies lignin content in the cell wall, reducing cadmium tolerance in *Arabidopsis thaliana* [20], while *miR159* modulates stomatal sensitivity to abscisic acid (ABA) and regulates drought response by targeting the *MYB33* gene [21].

## 2. Results

### 2.1. Phenotypic Analysis and Physiological Response to Low-Temperature Stress in Walnut Plants

**Figure 1:**
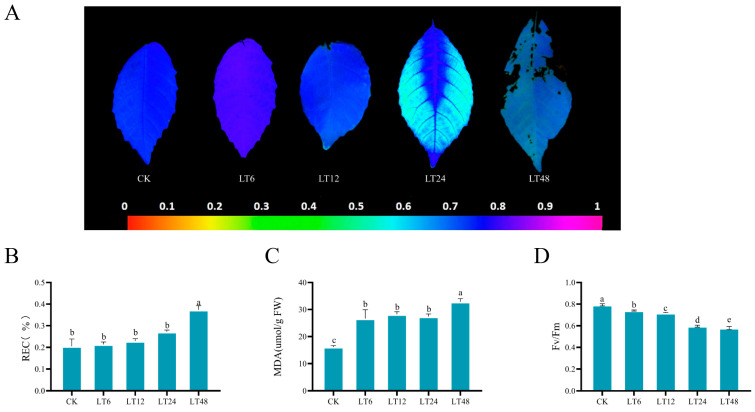
The effects of low temperatures at different time periods on the photosynthetic system and membrane stability of walnut leaves. (**A**) Changes in chlorophyll fluorescence Fv/Fm of walnut leaves after low-temperature stress at 4 °C; (**B**) electrolyte leakage; (**C**) MDA content in walnut leaves; (**D**) Fv/Fm values. Different letters indicate significant differences.

After 48 h of low-temperature treatment at 4 °C, walnut plants exhibited wilting and leaf necrosis. Chlorophyll fluorescence imaging of post-stress leaves revealed that the outer edge of the leaves was subjected to heavier stress. Changes in the Fv/Fm plots indicated that with increasing stress, the maximal photosynthetic efficiency of PSII was damaged to varying degrees, reaching destructive levels at 48 h (Figure 1A). The relative electrical conductivity (REC) of the plants gradually increased over time after low-temperature stress treatment, indicating damage to the cell membrane permeability. Malondialdehyde (MDA), a marker of membrane lipid peroxidation, was elevated and maintained at a certain concentration for 24 h after stress, peaking at 48 h (Figure 1B–D).

### 2.2. Transcriptome Data Processing

To investigate the key genes related to low-temperature stress in walnuts, leaves of walnut seedlings were sampled for five graded cryogenic treatment times, and three replicates were set up in each group. The samples underwent transcriptome sequencing. After eliminating reads containing adapters and low-quality reads, a total of 96.75 Gb of clean data was obtained. Each sample yielded 6.28 Gb of clean data, and the proportion of Q30 bases was above 95.21%. These results suggest that the RNA-Seq data exhibit high quality and are suitable for further analysis. Subsequently, 323,029,931 clean reads were mapped to the reference genome of walnut, Genome assembly Walnut 2.0. It was discovered that the mapping efficiency of the reads from each sample to the reference genome ranged from 94.93% to 95.55%.

**Figure 2:**
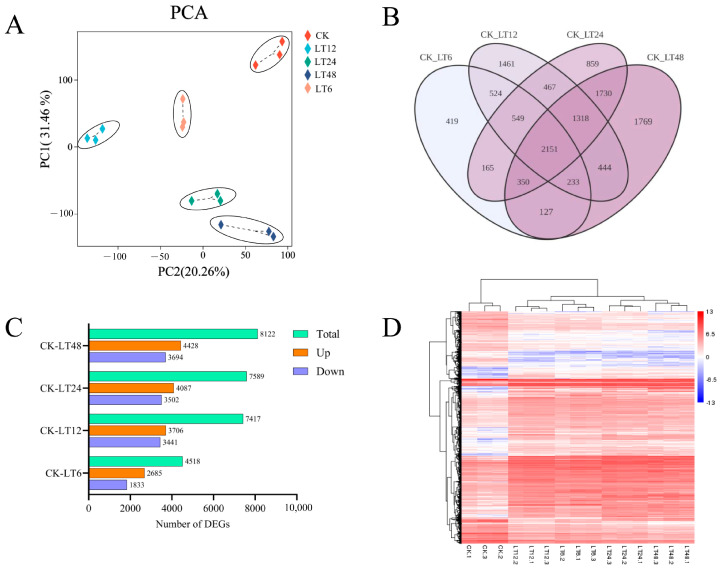
The impact of low-temperature stress on the differentially expressed genes (DEGs) in walnut leaves under control (CK) conditions compared to treatments LT6, LT12, LT24, and LT48. (**A**) Principal component analysis (PCA) of different samples; (**B**) Wayne plots of DEGs between different treatments and CK; (**C**) number of differential genes and number of up-regulated and down-regulated genes between treatment groups; (**D**) heatmap of expression clustering among DEGs of different treatments.

In order to effectively visualize the variations in the transcriptome data, a principal component analysis (PCA) was performed (Figure 2A). The results of the PCA exhibited a clear separation between the control (CK) and treatment groups. The first principal component accounted for 31.46% of the total variance, distinguishing different tissues significantly. The second principal component accounted for 20.26% of the total variance.

### 2.3. Differential Gene Expression

The analysis of differentially expressed genes at 6 h, 12 h, 24 h, and 48 h of low-temperature treatment identified 4518, 7147, 7589, and 8122 differentially expressed genes, respectively. A total of 12,566 differentially expressed genes were identified in each experimental group when compared with the transcriptome data of the control group (CK). Specifically, at CK_vs_LT6, 2685 up-regulated and 1833 down-regulated differentially expressed genes were detected; at CK_vs_LT12, 3706 were up-regulated and 3441 down-regulated; at CK_vs_LT24, 4087 were up-regulated and 3502 down-regulated; and at CK_vs_LT48, 4428 were up-regulated and 3694 down-regulated (Figure 2B). At 6 h of low-temperature stress, the number of differentially expressed genes was lower than that at other times and the number of up-regulated genes was higher than that of down-regulated genes; the number of differentially expressed genes started to decrease after 12 h, 24 h, and 48 h of treatment, and most of the increased differentially expressed genes were up-regulated. The four comparison groups had a total of 2151 differential genes, including 1342 up-regulated and 749 down-regulated genes (Figure 2C), and a heatmap shows the expression of these 2151 differential genes (Figure 2D).

### 2.4. GO and KEGG Enrichment Analysis

**Figure 3:**
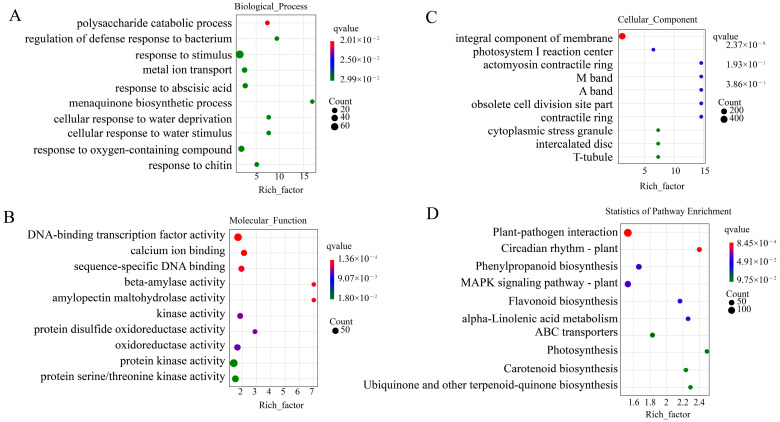
Gene Ontology (GO) enrichment analysis and Kyoto Encyclopedia of Genes and Genomes (KEGG) enrichment analysis bubble plots of the 2151 DEGs shared by the four groups CK_vs_LT6, CK_vs_LT12, CK_vs_LT24, and CK_vs_LT48. (**A**) Biological process. (**B**) Molecular function. (**C**) Cellular component. (**D**) KEGG statistics of pathway enrichment.

To better understand the function of these DEGs in response to low-temperature stress, Gene Ontology (GO) and Kyoto Encyclopedia of Genes and Genomes (KEGG) annotation analyses were performed. By performing GO analysis of the 2151 differential genes shared between groups and selecting the GO terms for the top ten biological processes that were most significantly enriched, it was found that the functions of these genes associated with low-temperature stress were mainly in response to abscisic acid (GO:009737), cellular response to water deprivation (GO:009414), response to oxygenated compounds (GO:1901701), and response to stimulus (GO:0050896). Among the DEGs common among the groups analyzed for KEGG pathway enrichment, the most significantly enriched pathways were phytopathogen interactions (ko04626), circadian rhythms (ko04712), phenylpropane biosynthesis (ko00940), plant MAPK signaling pathway (ko04010), flavonoid biosynthesis (ko00941), α-linolenic acid metabolism, ABC transporter protein (ko02010), and photosynthesis (ko00195) (Figure 3) (The GO and KEGG analyses among various groups are detailed in Supplementary File S1).

### 2.5. miRNA Characterization and Identification

**Figure 4:**
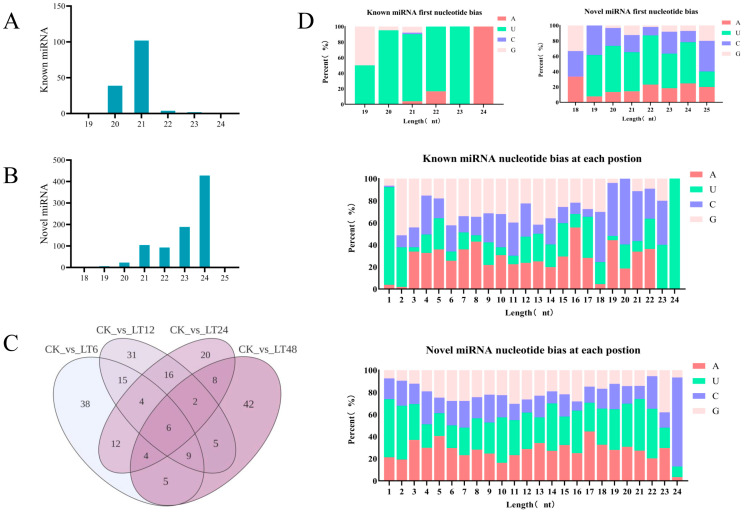
(**A**,**B**) Distribution of known as well as newly predicted miRNA lengths; (**C**) Wayne plots of differentially expressed miRNAs between groups; (**D**) miRNA base bias analysis.

Small RNA sequencing of another 15 samples yielded a total of 244.34 M clean reads from high-throughput sequencing, with no less than 10.44 M clean reads between each sample. The base quality value of Q30 ≥ 85%. A total of 1052 miRNAs were obtained for all samples, including 158 known miRNAs and 894 newly predicted miRNAs. Due to the specificity of the Dicer enzyme and DCL enzyme, the length distribution of the final generated mature miRNAs ranged from 19 nucleotides (nt) to 24 nt. Of these, most of the known miRNAs identified were 21 nt in length, followed by 20 nt, with only a very small fraction of other lengths. Most of the lengths of the newly predicted miRNAs are concentrated at 24 nt, with some distributions at 21 nt, 22 nt, and 23 nt (Figure 4A,B).

### 2.6. miRNA Family and Base Preference Analysis

The 1052 miRNAs were identified in 15 libraries. These miRNAs were categorized into 94 families, of which *miR171_1* was the largest with 36 members, followed by the *miR9672* family. The Dicer enzyme and DCL enzyme have a strong bias for the first base pair U at the 5′ end when recognizing and cleaving precursor miRNAs. From Figure 4D, it can be seen that the first base of microRNA at 21 nt has a strong preference for U and is resistant to C. The newly predicted microRNA pairs also prefer U at 24 nt, and are more evenly balanced for the other A, C, and G bases. Statistical microRNA base usage preferences are the basis for examining data quality.

### 2.7. Differential Expression and Functional Analysis of miRNAs

Many miRNAs showed significant differential expressions, and the Venn diagram of differentially expressed miRNAs between groups is shown in Figure 4. There are 34 differentially expressed miRNAs (DEMs) between group CK_vs_LT6 and group CK_vs_LT12, 26 DEMs between group CK_vs_LT6 and group CK_vs_LT24, 24 DEMs between group CK_vs_LT6 and group CK_vs_LT48, 28 DEMs between group CK_vs_LT12 and group CK_vs_LT24. There were a total of 22 DEMs between the CK_vs_LT12 group and the CK_vs_LT48 group, a total of 20 DEMs between the CK_vs_LT24 group and the CK_vs_LT48 group, and a total of 6 DEMs between the four groups (Figure 4C).

### 2.8. Joint Transcriptome and miRNA Analysis

To gain a more in-depth understanding of the regulatory role of microRNAs (miRNAs) in walnuts’ response to low-temperature stress, we carried out a prediction of the target genes of differentially expressed miRNAs (DEMs). In total, 7043 potential target genes were predicted from 217 DEMs. Subsequently, through a comprehensive co-analysis of all differentially expressed genes (DEGs) in the transcriptome with these predicted target genes, a total of 244 overlapping DEGs were identified.

**Figure 5:**
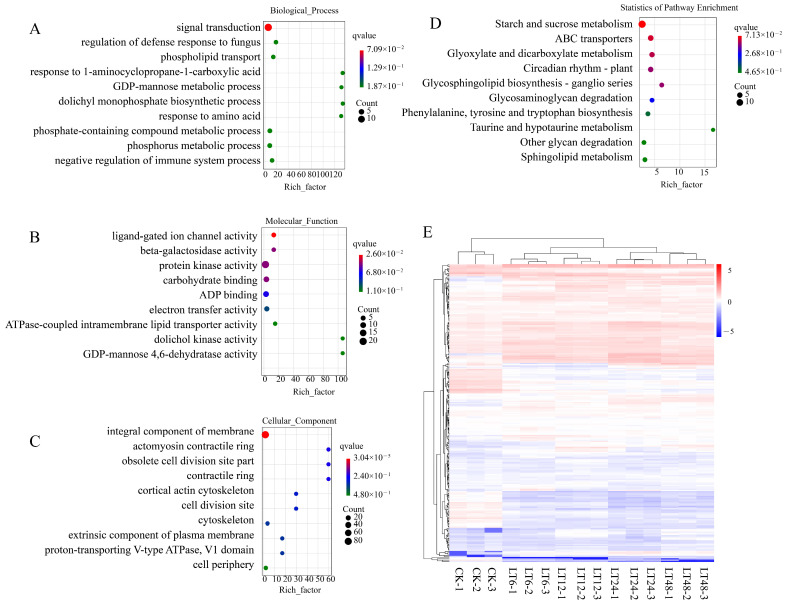
Bubble diagram of Gene Ontology (GO) enrichment analysis and Kyoto Encyclopedia of Genes and Genomes (KEGG) enrichment analysis of DEGs after co-analysis. (**A**) Biological process (**B**) Molecular function. (**C**) Cellular component. (**D**) KEGG statistics of pathway enrichment. (**E**) Heatmap of expression clustering of co-analyzed DEGs.

A cluster analysis heatmap was constructed for these 244 DEGs. The results of the heatmap analysis indicated that, when compared to the control group (CK), a greater number of genes exhibited up-regulated expression patterns following low-temperature stress, while only a small subset of genes showed down-regulated expression (Figure 5E). MicroRNAs generally exert negative regulatory effects on gene expression at the post-transcriptional level through complementary base-pairing with target messenger RNAs (mRNAs), which can lead to mRNA degradation or translational inhibition. We analyzed the expression levels of differentially expressed miRNAs (DEMs) and differentially expressed genes (DEGs). Initially, we identified a potential negative regulatory relationship in expression levels between 15 DEGs and 10 DEMs.

It is well recognized that a single miRNA can concurrently regulate multiple target mRNAs, and conversely, a single target mRNA can be regulated by multiple miRNAs. In our dataset, *novel_miR_436* potentially exerts negative regulatory effects on five genes, namely *gene-LOC108983866*, *gene-LOC108993086*, *gene-LOC108995409*, *gene-LOC109000913*, and *gene-LOC109011111*. Similarly, *novel_miR_275* may negatively regulate *gene-LOC108985086* and *gene-LOC109004668*.

When the expression level of *miR_436* increased from 6 h to 12 h, the expression levels of its corresponding five target mRNAs decreased simultaneously. Likewise, when the expression level of *miR_275* increased from 6 h to 48 h, the expression levels of its two corresponding target mRNAs also decreased concurrently. These findings may serve as focal points for our future research.

### 2.9. GO and KEGG Enrichment Analysis of Co-Analyzed DEGs

To better understand the function of these DEGs in response to low-temperature stress, we performed Gene Ontology (GO) Kyoto Encyclopedia of Genes and Genomes (KEGG) annotation analysis (Figure 5A–D). GO analysis was performed over these 244 DEGs to select the top ten most significantly enriched GO terms for biological processes, and these genes were found to be significantly enriched in signal transduction (GO:0007165), defense response to fungi (GO:1900150), and phospholipid transport (GO:0045332). And these genes were significantly enriched in the starch and sucrose metabolism (ko00500), ABC transporter (ko02010), and glyoxylate and dicarboxylate lipid metabolism (k00630) pathways. Therefore, we hypothesized that these DEGs may be a major part of the transcriptional response to cold stress.

### 2.10. Gene Expression Analysis in Starch and Sucrose Metabolism Pathway

**Figure 6:**
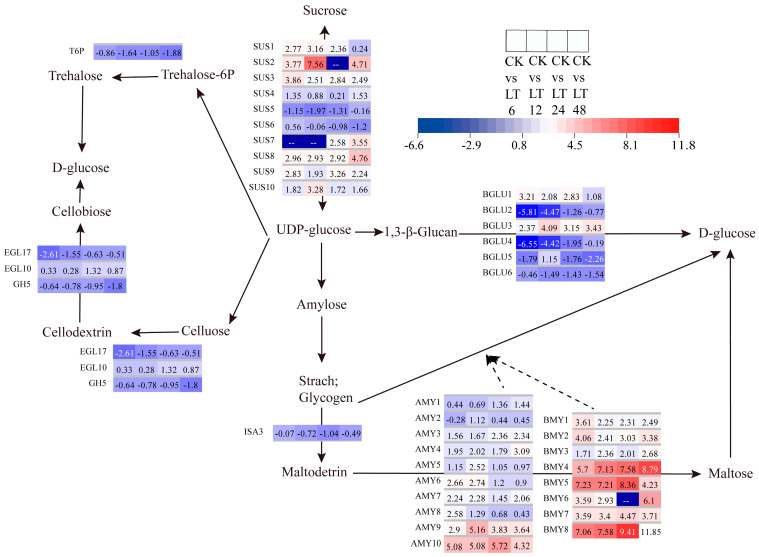
Heatmap of annotated genes in the starch and sucrose metabolism pathway. Solid lines and arrows represent biological processes and directions; dashed lines and arrows indicate that genes in this group also function in another process.

Starch and sucrose are the main forms of carbohydrates found in plants, exerting crucial roles in plants’ resistance to low-temperature (LT) stress as well as the maintenance of their growth and development. Based on an analysis of the functions of the transcriptome and miRNAs within the starch and sucrose metabolic pathways, the following conclusions are presented in this study (Figure 6). During the process of sucrose hydrolysis, specific genes annotated as sucrose synthase (*SUS*) including *SUS5*, *SUS2*, *SUS1*, and *SUS10* reached their peak expression levels at 12 h, whereas other sucrose synthase genes such as SUS4, *SUS8*, *SUS6*, and *SUS7* reached their peaks at 48 h. Furthermore, *SUS3* and *SUS9* reached their maximum expression levels at 6 h and 24 h, respectively. These up-regulated genes suggest that the key genes regulating sucrose hydrolysis undergo significant dynamic changes during LT stress treatment. During the process of starch hydrolysis, this study identified 10 α-amylase genes (*AMY1*–*AMY10*) and 8 β-amylase genes (*BMY1*–*BMY8*). These genes demonstrated significant expression at all time points during LT treatment, and as the stress duration prolonged, the expression levels of most genes exhibited an upward trend, further substantiating the close correlation between starch hydrolysis and LT stress response. During the process of cellulose hydrolysis, this study detected and analyzed the expression of two endoglucanase genes (*EGL10*, *EGL17*) and one glycosyl hydrolase gene (*GH5*). Among them, *EGL10*, being an up-regulated gene, reached its peak expression at 24 h, whereas *EGL17*, being a down-regulated gene, demonstrated the lowest expression at 6 h. Additionally, *GH5* also exhibited down-regulated expression at 48 h, although it reached a relatively high level. Within the trehalose synthesis pathway, this study observed the down-regulation of a gene, *T6P*, indicating that trehalose may also play a role in the response of walnuts to LT stress. In summary, the gene expression regulating starch and sucrose metabolic pathways changes with the LT stress process, indicating that LT treatment has a significant impact on the energy source distribution in walnuts.

### 2.11. Analysis of Differentially Expressed miRNAs (DEMs) Associated with Starch and Sucrose Metabolic Pathways and Their Target Genes Under LT Stress

**Figure 7:**
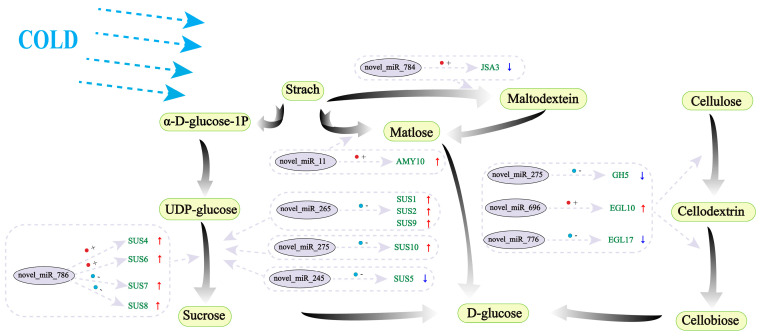
Expression relationship between DEMs and DEGs in the starch sucrose pathway under LT stress. Green boxes with black text represent compounds, purple boxes with black text represent DEMs, green text represents DEGs, red circles indicate positive regulation, blue circles indicate negative regulation, red arrows indicate up-regulation of target genes, and blue arrows indicate down-regulation of target genes.

Through in-depth analysis of DEGs and their corresponding DEMs in the starch and sucrose metabolic pathways of walnut leaves under low-temperature (LT) stress, we uncovered a series of intriguing regulatory mechanisms (Figure 7). These mechanisms elucidate how walnut leaves respond to LT stress by modulating sucrose synthesis, starch decomposition, and cellulose catabolism. In sucrose synthesis, multiple genes encoding sucrose synthase (*SUS1*, *SUS2*, *SUS9*) exhibited an up-regulation trend, indicating an enhanced sucrose synthesis capacity in walnut leaves under LT stress. Notably, these up-regulated sucrose synthase genes were negatively regulated by specific DEMs (*novel_miR_265*), while other DEMs (*novel_miR_786)* exhibited both positive and negative regulatory effects on different sucrose synthase genes. This complex regulatory pattern may facilitate the fine-tuning of sucrose synthesis rates to adapt to osmotic pressure changes under LT stress. In the starch decomposition pathway, the up-regulated α-amylase gene *AMY10* was positively regulated by *novel_miR_11*, potentially promoting rapid starch decomposition to provide energy and substrates for sucrose synthesis or other metabolic processes. Simultaneously, the down-regulated isoamylase gene *ISA3* was positively regulated by *novel_miR_784*, suggesting a selective inhibition of certain components of the starch decomposition pathway in walnut leaves under LT stress. In the cellulose catabolism pathway, down-regulation of the glycosyl hydrolase gene *GH5* may reduce the rate of cellulose decomposition, while up-regulation of the endoglucanase gene *EGL10* may promote localized degradation of cellulose to release monosaccharides such as glucose for energy metabolism. These two processes are regulated by *novel_miR_275* and *novel_miR_696*, respectively. Additionally, down-regulation of the gene *EGL17*, negatively regulated by *novel_miR_776*, may further influence the activity of the cellulose catabolism pathway. In summary, walnut leaves employ a sophisticated miRNA-mRNA regulatory network to finely tune sucrose synthesis, starch decomposition, and cellulose catabolism under LT stress, enabling them to adapt to stressful environments and maintain normal physiological functions. These findings not only enhance our understanding of the LT stress response mechanisms in walnut leaves but also provide potential targets for future gene engineering efforts to improve plant stress resistance.

### 2.12. qRT-PCR Validation

**Figure 8:**
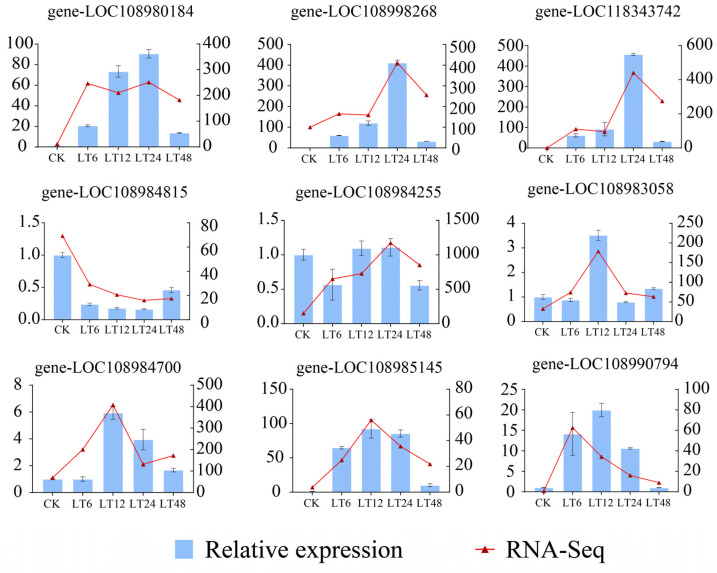
Verification of candidate differentially expressed genes (DEGs) through qRT-PCR. The blue bar chart represents the expression levels of DEGs in qRT-PCR, while the red line chart indicates the expression levels of DEGs in the transcriptome.

To verify the reliability of the transcriptome sequencing results, quantitative real-time polymerase chain reaction (qRT-PCR) analysis was employed. In this study, a total of nine co-analyzed differentially expressed genes (DEGs) were randomly selected and subjected to examination via qRT-PCR. The transcriptome sequencing results were compared with the results of qRT-PCR experiments. Our results show that almost all expression levels analyzed by qRT-PCR are highly consistent with the transcriptome sequencing results, even if there is a mismatch in the fold change in the expression levels of some genes detected by transcriptome sequencing and qRT-PCR analysis. These results also confirm the reliability of the transcriptome sequencing data (Figure 8) (The primers for qRT-PCR genes are detailed in Supplementary File S2).

## 3. Discussion

Abiotic stresses such as drought, salinity, and low temperature are major constraints limiting the development of the walnut industry. Investigating the response mechanism of walnuts under abiotic stress conditions is crucial for screening and breeding resistant and high-yielding varieties. However, limited research has been conducted on this aspect of low-temperature stress in walnuts.

In this study, experiments were conducted by subjecting walnut plants to different time periods of low-temperature treatment. It has been shown that under light conditions, photosystem II (PS II) is more severely damaged with increasing stress duration [22]. The attenuation of the maximum quantum yield Fv/Fm determines the potential maximum photosynthetic efficiency decline of PSII, demonstrating that PSII’s damage is due to low-temperature stress. Similar results were found in our experiments, from an initial lack of significant change from the CK after 12 h of treatment to significant damage after 24 h of treatment, and finally a gradual necrosis of the marginal parts of the leaves starting after 48 h of treatment. REC is negatively correlated with plant plasma membrane integrity and is an important indicator of plant response to stress [23]. MDA is a marker that measures lipid peroxidation in plant cells and is commonly used to assess stress tolerance in plants [24]. REC and MDA are important indicators in measuring membrane damage and cellular stability [25]. The REC and MDA of leaves gradually increased with time as the stress increased, maintaining a steady state between 6 h and 24 h and peaking at 48 h.

RNA-seq analysis revealed 4518, 7147, 7589, and 8122 *DEGs* at 6 h, 12 h, 24 h, and 48 h of low-temperature treatment, respectively. KEGG enrichment analysis showed that many cold-responsive genes are involved in a variety of biological processes, The main topics include phenylpropane biosynthesis, the MAPK signaling pathway, flavonoid biosynthesis, ABC transporter proteins, and photosynthesis.

Small RNA sequencing showed that the number of miRNA lengths of 24 nt was the highest among all groups of miRNA lengths, which is highly consistent with previous studies on *Arabidopsis thaliana* [26], *Oryza sativa* [27], *thistledown alfalfa* [28], and *woolly poplar* [29]. Known miRNAs were analyzed, new miRNAs were predicted, and miRNA families were classified, with *miR171_1* being the most abundant family of miRNAs in response to low-temperature stress. miR171 has an important role in plant response to abiotic stress. *MSTRG.28732.3* interacts with miR171 through the chlorophyll biosynthesis pathway, affecting drought tolerance in rice by regulating *Os02g0662700*, *Os02g0663100* and *Os06g0105350* [30]. miR171 is also responsive to multiple abiotic stresses in *Arabidopsis thaliana* [31]; we therefore hypothesized that miR171 may play an important role in the response of walnut to low-temperature stress.

Numerous studies have consistently demonstrated that exposure to low-temperature stress triggers the accumulation of soluble sugars in plants, subsequently enhancing their tolerance to such stress conditions [32,33]. Analysis of the differentially expressed genes (DEGs) associated with starch and sucrose metabolic pathways demonstrated that the expression patterns of endoglucanase genes (*EGL10*, *EGL17*) and the glycosyl hydrolase gene (*GH5*) during cellulose hydrolysis, as well as the expression of the trehalose-6-phosphate synthase (*T6P*) gene in the alginate synthesis pathway, were in line with the previously reported results [34,35]. Additionally, low temperatures have been shown to elevate the activity levels of key enzymes involved in sucrose metabolism, including sucrose phosphate synthase (*SPS*), sucrose synthase (*SUS*), and invertase (*INV*), ultimately leading to an increase in sucrose levels [36]. A comprehensive analysis of DEGs in the starch–sucrose pathway identified a total of 11 sucrose synthase genes, with the majority of them exhibiting up-regulation. This finding suggests that low-temperature treatments stimulate the synthesis of sucrose in walnut leaves, thereby enhancing the content of soluble sugars as a stress response mechanism.

Starch constitutes the primary form of carbohydrate storage in plants and serves as a pivotal molecule in mediating plant responses to cold stress [37]. Numerous studies have demonstrated that plants exposed to cold stress enhance their tolerance through the degradation of starch. Furthermore, functional analyses of these genes have revealed that specific isoforms of the β-amylase (*BMY*) gene exhibit cold-activation properties [38]. *PtrBAM1*, a β-amylase, confers resistance to cold stress by modulating the levels of soluble sugars, which function as osmolytes and antioxidants [39]. The overexpression of *PbrBAM3* in tobacco (*Nicotiana tabacum*) and pear (*Pyrus ussuriensis*) facilitates starch degradation and augments cold tolerance following cold treatment [40]. When synergized with BMY, AMY exhibits effective starch degradation capabilities [41]. Arabidopsis mutants lacking *AMY3* and *BMY1* exhibit heightened sensitivity to osmotic stress and accumulate reduced levels of soluble sugars and proline under stress conditions [42]. In the present study, a total of 10 *AMY* genes were identified among DEGs associated with cold stress, along with eight *BMY* genes, all of which were up-regulated following cold stress exposure. These findings indicate that *AMY* and *BMY* may individually or synergistically regulate starch degradation in walnuts under cold stress conditions, thereby enabling a response to stress.

Plants respond to stress conditions by synthesizing numerous defense compounds [43]. A significant proportion of these compounds exist in glycosylated form, with glycosylation enhancing their solubility and stability, thereby enabling their storage in vesicles or other organelles and further safeguarding the plant from the detrimental impacts of stress on its defense mechanisms [44,45]. Beta-glucosidase (*BGLU*) constitutes an important class of enzymes responsible for activating these compounds through glycosylation. β-Glucosidase catalyzes the hydrolysis of terminal non-reducing β-D-glucosyl residues, thereby releasing β-D-glucose [46]. The *BGLU* gene plays a crucial role in the activation of defense compounds in response to stress conditions and in the liberation of active phytohormones [47]. Under dehydration stress, *AtBG1* and *AtBG2*, encoded by the Arabidopsis β-glucosidase genes *AtBGLU18* and *AtBGLU33*, respectively, exhibited ABA-GE hydrolytic activity, thereby functioning to elevate ABA levels [48,49]. The increased expression and enzyme activity of these genes allowed the plants to rapidly augment their ABA levels. In rice, *Os3BGLU6* exhibited responsiveness to drought and ABA treatments. Disruption of *Os3BGLU6* in rice led to decreased ABA content and increased stomatal density [50]. In this study, we identified a total of six *BGLU* genes, including two that were up-regulated and four that were down-regulated. The majority of these genes exhibited peak expression at 6 h and 12 h during the early stages of stress, suggesting that β-glucosidase plays a significant role in the initial response of walnuts to low-temperature stress.

## 4. Materials and Methods

### 4.1. Materials

The experimental material was the Xinjiang ‘Wen 185’ walnut variety.

### 4.2. Test Methods

The experiment was conducted at the Agricultural Science Building, Faculty of Agriculture, Shihezi University, where ‘Wen 185’ walnut seeds from the previous year were collected, placed in a refrigerator for storage at 4 °C for 1 week, and then sown in nutrient pots (containing charcoal soil/vermiculite = 3:1). The culture temperature was set at 25 °C, and the humidity was maintained at 70%. The seeds were then incubated with a 16/8 h (light/dark) photoperiod at a light intensity of 80 µmol·m^−2^·s^−1^. When the seedlings grew to 5 compound leaves, the well-grown and consistent seedlings were screened for 4 °C low-temperature stress, and 2–5 functional leaves under the top page were taken at 0 h, 6 h, 12 h, 24 h, and 48 h, respectively, put into the freezing tube with liquid nitrogen for rapid freezing, and then placed in a −80 °C refrigerator for preservation. Three biological replicates were set up for each treatment period. Samples were sequenced and analyzed by Biomarker Technologies (Wuhan, China).

### 4.3. Measurement Indicators and Methods

Relative electrical conductivity (REC) and malondialdehyde (MDA) levels were determined using conductivity and thiobarbituric acid methods, respectively [51].

### 4.4. RNA Extraction

Library construction and sequencing were performed following standard protocols. Total plant RNA was extracted using RNAprep Pure Plant Kit (Tiangen, Beijing, China). RNA concentration and purity were measured using a NanoDrop 2000 spectrophotometer (Thermo Fisher Scientific, Wilmington, DE, USA). To guarantee the use of qualified samples for transcriptome sequencing, a NanoDrop 2000 spectrophotometer (Thermo Fisher Scientific, Wilmington, DE, USA) was used; to detect the purity and concentration of RNA, an Agient 2100/LabChip GX assay(Agilent Technologies, Santa Clara, CA, USA) was used. After the samples were tested, eukaryotic mRNA was enriched with magnetic beads with Oligo (dT), and the mRNA was randomly interrupted by the addition of Fragmentation Buffer. Using mRNA as a template, the first cDNA strand and second strand were synthesized, and cDNA purification was carried out. The purified double-stranded cDNA was then subjected to end repair, addition of A-tails, and ligation of sequencing junctions, and then fragment size selection was carried out by using AMPure XP beads(Beckman Coulter, Brea, CA, USA), and finally the cDNA library was obtained by PCR enrichment. After the library quality control was performed, PE150 mode sequencing was performed using a high-throughput sequencing platform. After the sequencing data were downloaded, They were analyzed using the bioinformatics analysis process provided by the BMK—Cloud platform (www.biocloud.net URL accessed on 1 July 2024). The raw reads were filtered, and the clean reads were mapped to the reference sequence using HISAT 2.2.4 software. The gene expression levels were calculated using the fragments per kilobase per million mapped reads (FPKM) method. Finally, the DESeq2 software version 1.30.1 was used to determine the differential RNA expression between the groups using the criteria of *p* < 0.05|log-fold change| ≥ 2 to identify the differentially expressed genes (DEGs). The Gene Ontology (GO) and Kyoto Encyclopedia of Genes and Genomes (KEGG) enrichment analyses of the DEGs were performed using R version 2.48.0 based on the hypergeometric distribution. The GO terms and KEGG pathways with *p* < 0.05 were considered significantly enriched. The downstream data were filtered to obtain the mapping data and sequence-matched against the specified reference genome.

### 4.5. Screening for Differentially Expressed Genes

The sample fractions were divided into CK_LT6, CK_LT12, CK_LT24, and CK_LT48, and differential fold change (Fold change) ≥ 2 and false discovery rate (FDR) < 0.01 were used as the screening criteria for differentially expressed genes (DEGs).

### 4.6. Small RNA Library Construction and Small RNA Sequencing

The NEBNext Multiplex Small RNA Library Preparation Kit (New England Biolabs, Inc., Hitchin, UK) was used for small RNA library construction according to the manufacturer’s instructions. A 10 µg amount of total RNA from 15 independent samples was ligated with 30 adapters and 50 adapters with T4 RNA ligase. A 10 µg amount of total RNA from 15 independent samples was ligated with 30 adapters and 50 adapters with T4 RNA ligase. Double-stranded cDNA was synthesized by reverse transcription of RNA using Superscript II reverse transcriptase. PCR amplification of DNA fragments was performed. The products were then separated by fragment size using a PAGE gel. Quality control of PCR-enriched fragments was performed using Agilent 2100 to detect fragment size and DNA library distribution. Total library concentration was assayed with Picogreen. These libraries were subjected to single-end sequencing on the Hi-Seq 2500 platform of Shanghai Personal Biotech (Shanghai, China).

### 4.7. Statistical Analysis of Data

Data were processed using Microsoft Excel 2016 and SPSS 19.0 for one-way ANOVA, with Duncan’s test for significance (*p* < 0.05). Graphs were generated using Origin 2022.

## Data Availability

All the data that support the findings of this study are available in this paper.

## Supplementary Materials

The supporting information can be downloaded at: https://www.mdpi.com/article/10.3390/ijms26041401/s1.
