# Supplementary figures and images for: Transcriptome and MicroRNA Analysis of Juglans regia in Response to Low-Temperature Stress
Source: Int J Mol Sci. 2025 Feb 7;26(4):1401. doi: 10.3390/ijms26041401 (PMC11855649; doi:10.3390/ijms26041401)

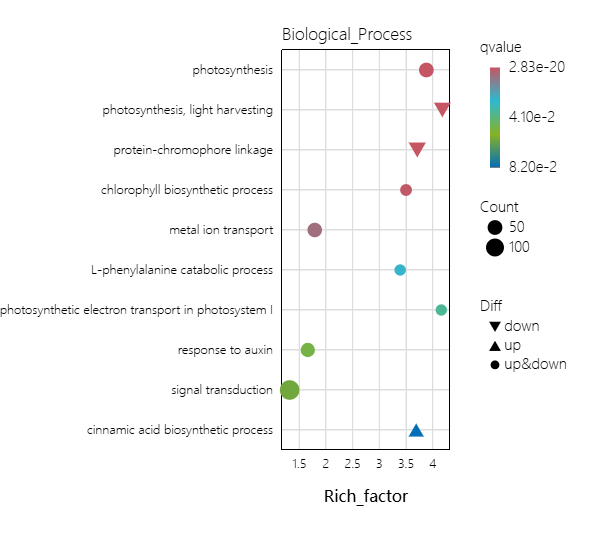

Supplement: Supplementary file 1 [file ijms-26-01401-s001.zip › Supplementary File 1/12/12h-GO-BP.png]

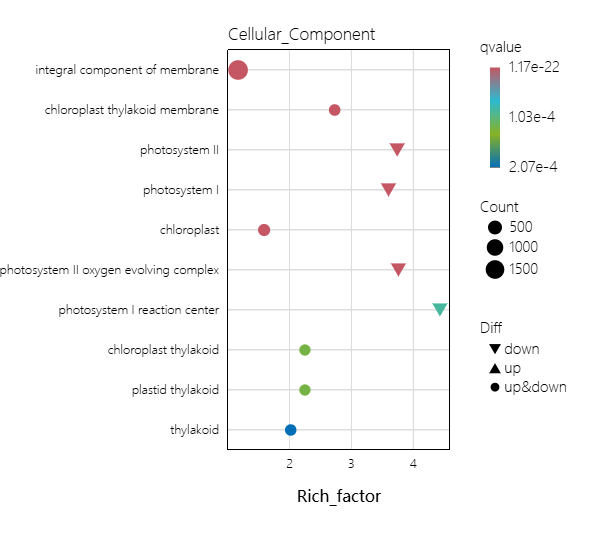

Supplement: Supplementary file 1 [file ijms-26-01401-s001.zip › Supplementary File 1/12/12h-GO-CC.png]

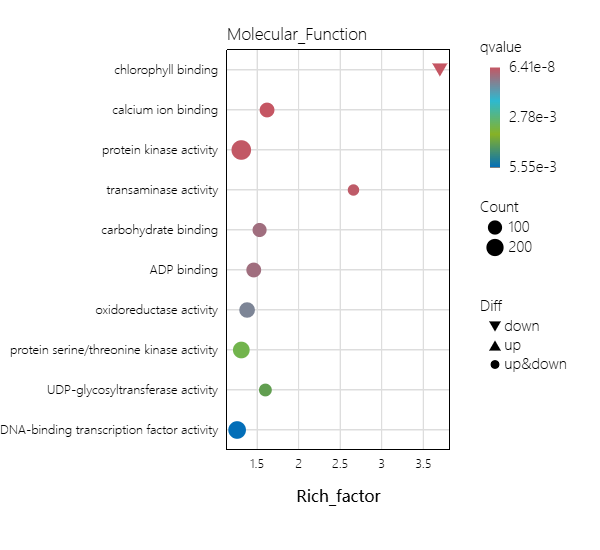

Supplement: Supplementary file 1 [file ijms-26-01401-s001.zip › Supplementary File 1/12/12h-GO-MF.png]

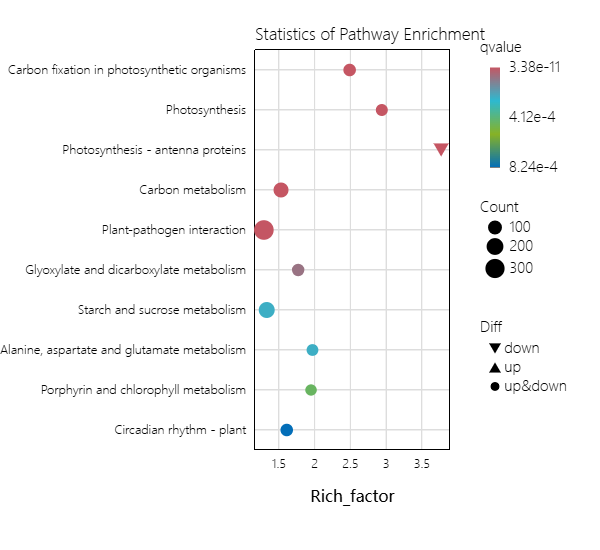

Supplement: Supplementary file 1 [file ijms-26-01401-s001.zip › Supplementary File 1/12/12h-KEGG.png]

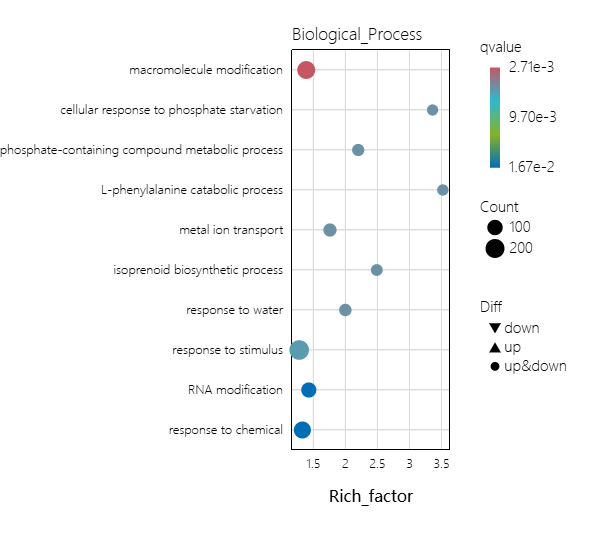

Supplement: Supplementary file 1 [file ijms-26-01401-s001.zip › Supplementary File 1/24/24h-GO-BP.png]

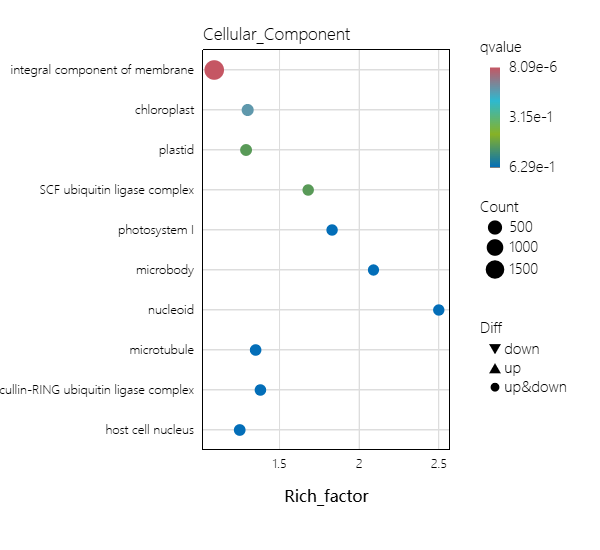

Supplement: Supplementary file 1 [file ijms-26-01401-s001.zip › Supplementary File 1/24/24h-GO-CC.png]

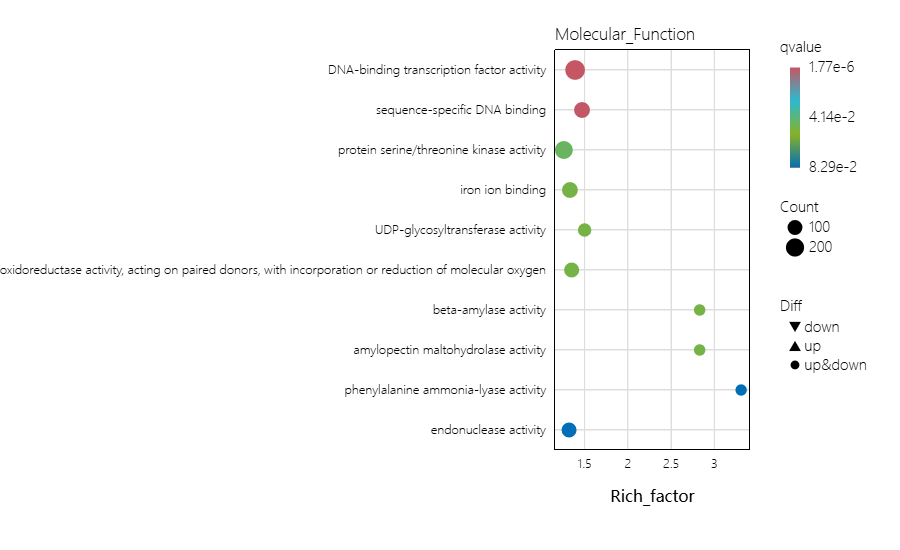

Supplement: Supplementary file 1 [file ijms-26-01401-s001.zip › Supplementary File 1/24/24h-GO-MF.png]

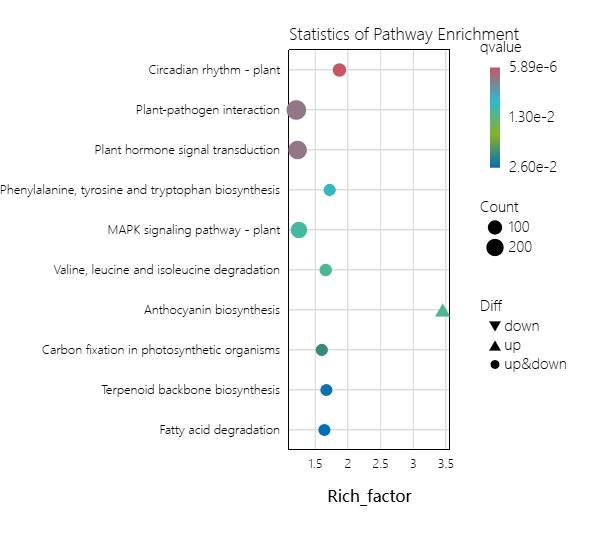

Supplement: Supplementary file 1 [file ijms-26-01401-s001.zip › Supplementary File 1/24/24h-KEGG.png]

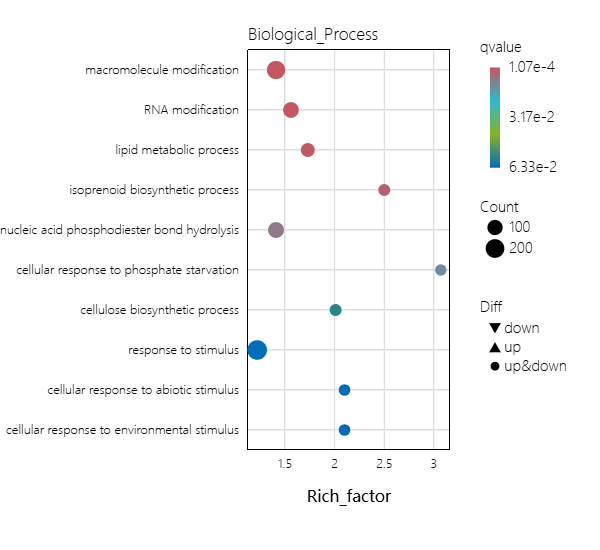

Supplement: Supplementary file 1 [file ijms-26-01401-s001.zip › Supplementary File 1/48/48h-GO-BP.png]

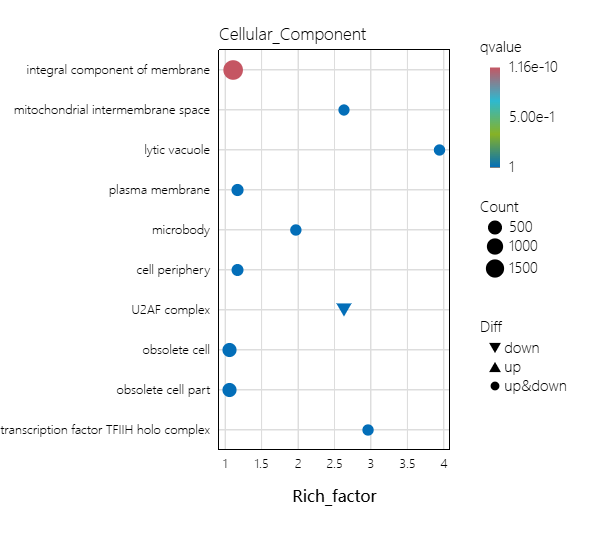

Supplement: Supplementary file 1 [file ijms-26-01401-s001.zip › Supplementary File 1/48/48h-GO-CC.png]

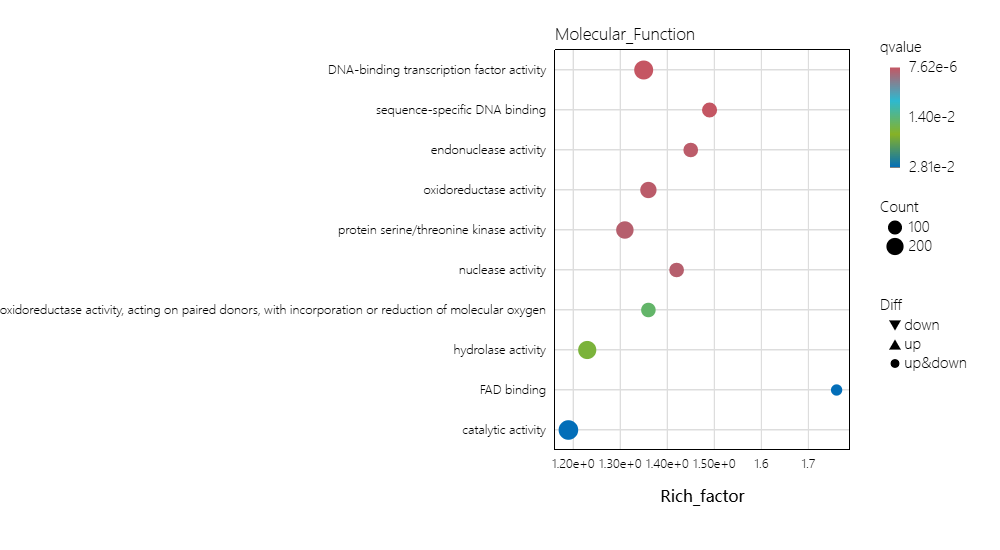

Supplement: Supplementary file 1 [file ijms-26-01401-s001.zip › Supplementary File 1/48/48h-GO-MF.png]

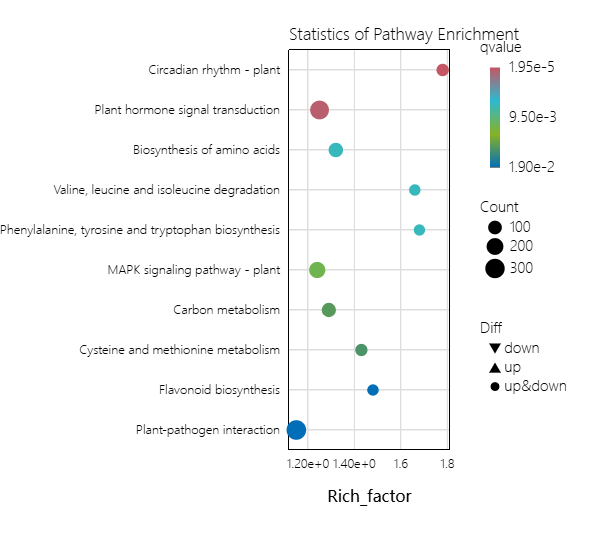

Supplement: Supplementary file 1 [file ijms-26-01401-s001.zip › Supplementary File 1/48/48h-KEGG.png]

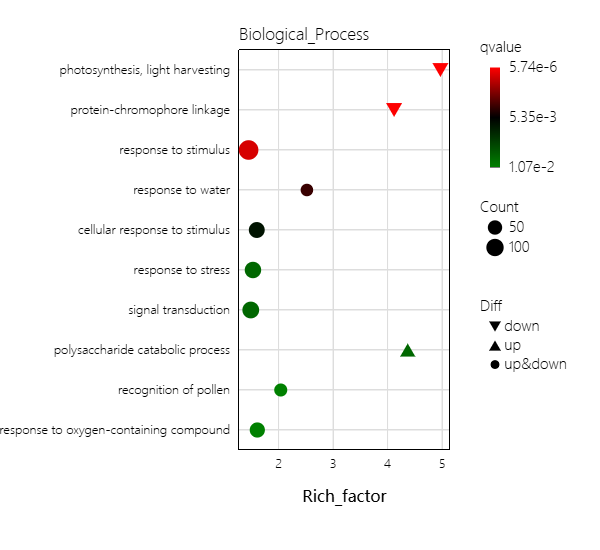

Supplement: Supplementary file 1 [file ijms-26-01401-s001.zip › Supplementary File 1/6/6h-GO-BP.png]

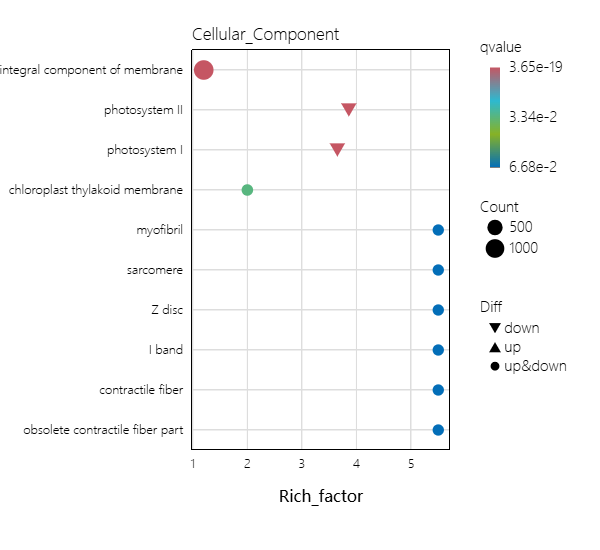

Supplement: Supplementary file 1 [file ijms-26-01401-s001.zip › Supplementary File 1/6/6h-GO-CC.png]

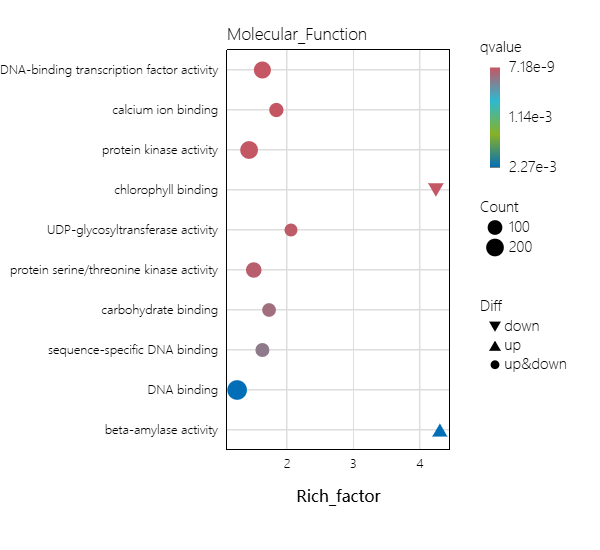

Supplement: Supplementary file 1 [file ijms-26-01401-s001.zip › Supplementary File 1/6/6h-GO-MF.png]

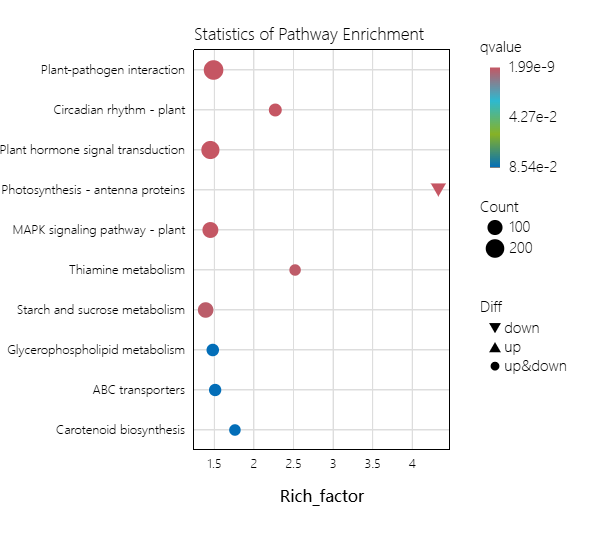

Supplement: Supplementary file 1 [file ijms-26-01401-s001.zip › Supplementary File 1/6/6h-KEGG.png]
